# The Reality behind Informal Health Payments in Iran: “Under the Table Payments” or “On the Table Payments”?

**Published:** 2017-02

**Authors:** Mohammad MESKARPOUR-AMIRI, Abbas ASSARI-ARANI, Hosein SADEGHI, Lotfali AGHELI

**Affiliations:** Dept. of Health Economics, Faculty of Management and Economics, Tarbiat Modares University, Tehran, Iran

## Dear Editor-in-Chief

Unfortunately, informal payments for health care are responsible for a considerable share of patients’ out-of-pocket payments in Iran (1, 2). Almost all health care users and providers are familiar with such payments with the name of “under the table payments” in Iran (3). About half of Iranian health care seekers have faced with such payments at least one time during a year (2).

Although the informal payments for health care is a common phenomenon in developing and in transition countries, but what made Iran’s condition a little different is the nature and reasons behind such payments. Informal payments in Iran are largely originated from government mal-intervention, health care system mal-function and some of our wrong cultures, *not health care provider’s corruption activities*. Physicians are objecting that the government-determined imperiously medical tariffs are very low and do not reflecting the differences in their experiences, skills, etc. Patients believe that physicians who receive informal payments are more experienced and skillful than others. In addition, our health system malfunction allows patients for receiving faster care from the over-need specialized physicians through paying extra payments (4). However, the share of informal payments for corruption and criminal practices-such as illegal abortion or opioids prescription- seems to be relatively low in Iran. Therefore, receiving and paying “under the table payments” in Iran health care system are so common and clear that we can call it “on the table payments”.

Finally, all informal payments, despite of their nature, can have same adverse effects on health system equity and efficiency. Therefore, the informal health payments mainly are not originated from criminal activities in Iran; we still strongly believe that health policy makers should pay all of their attention for reducing such payments in Iran.
